# Prognostic Significance and Gene Co-Expression Network of *PLAU* and *PLAUR* in Gliomas

**DOI:** 10.3389/fonc.2021.602321

**Published:** 2022-01-11

**Authors:** Junhong Li, Huanhuan Fan, Xingwang Zhou, Yufan Xiang, Yanhui Liu

**Affiliations:** ^1^ Department of Neurosurgery, West China Hospital of Sichuan University, Chengdu, China; ^2^ West China Brain Research Center, West China Hospital of Sichuan University, Chengdu, China

**Keywords:** *PLAU*, *PLAUR*, glioma, prognosis, gene network

## Abstract

The urokinase-type plasminogen activator(PLAU) and its receptor PLAUR participate in a series of cell physiological activities on the extracellular surface. Abnormal expression of *PLAU* and *PLAUR* is associated with tumorigenesis. This study aims to evaluate the prognostic value of *PLAU/PLAUR* transcription expression in glioma and to explore how they affect the generation and progression of glioma. In this study, online databases are applied, such as Oncomine, GEPIA, CGGA, cBioPortal, and LinkedOmics. Overexpression of PLAU/PLAUR was found to be significantly associated with clinical variables including age, tumor type, WHO grade, histology, IDH-1 mutation, and 1p19q status. *PLAU* and *PLAUR* had a high correlation in transcriptional expression levels. High expression of *PLAU* and *PLAUR* predicted a poor prognosis in primary glioma and recurrent glioma patients, especially in lower grade gliomas. Cox regression analysis indicated that high expression of *PLAU* and *PLAUR* were independent prognostic factors for shorter overall survival in glioma patients. In gene co-expression network analysis *PLAU* and *PLAUR* and their co-expression genes were found to be involved in inflammatory activities and tumor-related signaling pathways. In conclusion, PLAU and PLAUR could be promising prognostic biomarkers and potential therapeutic targets of glioma patients.

## Introduction

Glioma, a broad category of brain tumors with high fatality rate, is the most common type among primary malignant brain tumors in adults, though accounting for less than 1% of all newly diagnosed tumors (1). Among all kinds of diffuse glioma, 70-75% of them are glioblastoma(GBM), which is the most fatal one, with a median overall survival less than 2 years after standard chemoradiotherapy. Molecular therapy that targets epigenetic alterations is under evaluation and is supposed to lead to an important breakthrough in the treatment of malignancies like GBM (2). An increasing number of evidences suggest that the integrated histological-molecular classification may be superior to the traditional histological classification (3). Biomarkers like isocitrate dehydrogenase 1(IDH-1) and O6-methylguanine-DNA methyltransferase (MGMT) not only play a crucial role in diagnosis and prognosis but also work as potential therapeutic targets. A deeper research of these biomarkers provides an essential framework to treat particular glioma subtypes (4).

*PLAU* (plasminogen activator, urokinase), also known as *UPA*, encodes a selected serine protease that converts plasminogen to plasmin. PLAUR, the receptor of PLAU, is bound to cell membranes by a glycosyl phosphatidy linositol anchor, and plays an important role in localizing and promoting plasmin formation. The PLAU-PLAUR system participates in a variety of physiological and pathological processes of the non-malignant cells during embryogenesis, wound healing and post-lactational involution; and it is also involved in tumorigenesis such as angiogenesis and metastasis (5, 6). In recent years, more and more studies have discovered that PLAU/PLAUR system is both involved in chronic diseases like rheumatoid arthritis and Quebec platelet disorder, and in malignant diseases such as breast cancer, colorectal cancer, ovarian cancer, lung cancer, and melanoma, etc. (7–12). In addition to the abnormal expression during tumor progression, PLAU and PLAUR are also found involved in complicated tumor invasion and cell migration. These features result in biological more aggressive tumors and poor prognosis of patients (13, 14).

Previous studies about the relationship between glioma and PLAU/PLAUR have indicated that high expression of PLAU/PLAUR promotes glioma cell invasion, tumor growth, and angiogenesis (15, 16). Moreover, their mechanisms and pathways have been explored and reported these years, such as GRB2/AKT/BAD pathway, PI3k/AKT pathway, and other relevant mechanisms (17–20). Although the number of publications about PLAU/PLAUR in glioma has grown each year, less attention is paid to clinicopathological features and prognosis. In the mid-90s, Hsu et al. reported a correlation between high expression of *PLAU* and bad prognoses of glioma patients (21). And approximately 20 years ago, Zhang et al. came to the same results using Northern blot hybridization and immunohistochemical detection (22). Nowadays, as large datasets with molecular-genetic data from modern platforms are available, the mentioned survival analytic results with outdated methods and small samples are not representative any more.

Different kinds of online databases, tools and integrate data were applied in this study. First, the transcription expression level of *PLAU/PLAUR* among glioma patients were investigated. Then, their relations with clinical parameters were analyzed, while the prognostic factors were also analyzed. Furthermore, potential gene functions and pathways were predicted through data mining.

## Materials and Methods

### Ethics Statement

This study was approved by the Academic Committee of Sichuan University, and conducted according to the principles expressed in the Declaration of Helsinki. All the datasets were retrieved from the published literature, so it was confirmed that all written informed consent was obtained.

### Oncomine Database

Oncomine database (http://www.oncomine.org) is a cancer microarray database and integrated data-mining platform, consisting of 715 independent databases and 86733 tumor samples (23). In the current study, the Oncomine database was retrieved for the transcription expression of *PLAU* and *PLAUR* in different glioma tissues and adjacent normal brain tissues, and the differences in transcription expression were analyzed with students’ t-test. Cut-off of p-value and fold change were set as follows: p-value: 0.05, fold change: 2, gene rank: top 10%.

### GEPIA Database

Gene Expression Profiling Interactive Analysis (GEPIA) (http://gepia.cancer-pku.cn) is a newly developed interactive web server for analyzing the RNA sequencing and expression data of 9736 tumors and 8587 normal samples from The Cancer Genome Atlas (TCGA) and Genotype-Tissue Expression (GTEx), using a standard processing pipeline (24). Transcription expressions between gliomas and normal brain tissues were compared with GEPIA in this study, and a survival analysis in glioma patients with different WHO grades was also conducted.

### CGGA Database

Chinese Glioma Genome Atlas (CGGA) database (http://www.cgga.org.cn) is a web application for data storage and analysis (25). It explores brain tumors datasets of over 2,000 samples from Chinese cohorts. This database includes the whole-exome sequencing, DNA methylation, mRNA sequencing, mRNA microarray, and microRNA microarray and matched clinical data. Clinicopathological data was downloaded from the CGGA database. A survival analysis was performed on the selected glioma patients. And a Pearson correlation analysis was also carried out.

### cBioPortal

The cBio Cancer Genomics Portal (cBioPortal) (https://www.cbioportal.org) is a web for exploring, visualizing, and analyzing multidimensional cancer genomics data (26). Genomic data types integrated by cBioPortal include somatic mutations, DNA copy-number alterations, mRNA and microRNA expression, DNA methylation, protein abundance, and phosphoprotein abundance. The cBioPortal online tool was used for detecting the frequency of gene alterations and for survival analysis between the gene-altered group and unaltered group.

### LinkedOmics

LinkedOmics (http://www.linkedomics.org) is a publicly available portal that includes multi-omics data from all 32 TCGA Cancer types (27). This web application is consist of three analytical modules: LinkFinder, LinkInterpreter and LinkCompare. With LinkFinder, attributes relevant to a query attribute would be searched out, for example, mRNA or protein expression signatures of genomic alterations, candidate biomarkers of clinical attributes, and candidate target genes of transcriptional factors, microRNAs, or protein kinases. To get biological interpretation from the association results, an enrichment analysis is performed with the LinkInterpreter module based on Gene Ontology, biological pathways, network modules, among other functional categories. In the current study, LinkFinder and LinkInterpreter were used to explore the potential gene regulation network.

### Statistical Analysis

Statistic analyses were carried out using SPSS software(version 22.0, IBM Corp, New York, USA). Overall survival (OS) was defined from the time of surgery to death or last follow-up. Variables with normal distribution were analyzed with students’ t-test, otherwise by the Mann-Whitney U test. One-way analysis of variance was used in the case of more than 2 data sets. Cox regression analysis was used to assess the prognostic values of clinical factors based on the data from CGGA. Statistical significance was defined as two-sided p<0.05.

## Results

### *PLAU* and *PLAUR* Transcription Levels in Gliomas

Oncomine database was applied to investigate whether there were differences between glioma and normal brain tissue in the expression of the *PLAU* gene. As shown in **Table 1**, mRNA expressions of *PLAU* were significantly upregulated in 7 databases respectively, where 5 databases indicated overexpression of *PLAU* in GBM compared to normal brain tissue, the fold change ranged from 2.7 to 9.1 (28–31). In addition, *PLAU* mRNA overexpression in other kind of cells was also reported. The dataset of Gutman suggested *PLAU* mRNA overexpression in pilocytic astrocytoma with a fold change of 4.3 (32). And the Rickman’s dataset suggested it in astrocytoma with a fold change of 17.2 (33). With respect to the transcription expression of *PLAUR*, 3 databases were retrieved and indicated overexpression of PLAUR in GBM compared to normal brain tissue with fold changes of 2.4, 2.5 and 7.0 respectively (28–30).

**Table 1:** Significant changes of PLAU and PLAUR expression in transcription level between glioma and normal brain tissues (ONCOMINE).

Gene	Types of Glioma VS Normal brain	Fold Change	t-test	P-value	Dataset
**PLAU**	Glioblastoma	2.9	17.527	1.08E-16	TCGA
	Glioblastoma	2.7	7.743	9.39E-6	Liang Brain
	Pilocytic Astrocytoma	4.3	4.947	9.43E-4	Gutmann Brain
	Glioblastoma	3.6	8.328	2.99E-8	Lee Brain
	Glioblastoma	4.5	9.269	1.51E-12	Sun Brain
	Glioblastoma	9.1	4.360	0.006	Bredel Brain
	Astrocytoma	17.2	3.112	0.012	Rickman Brain
**PLAUR**	Glioblastoma	7.0	10.379	1.48E-11	Bredel Brain
	Glioblastoma	2.4	5.529	6.91E-4	Lee Brain
	Glioblastoma	2.5	3.081	0.032	Liang Brain

**Figure 1:**
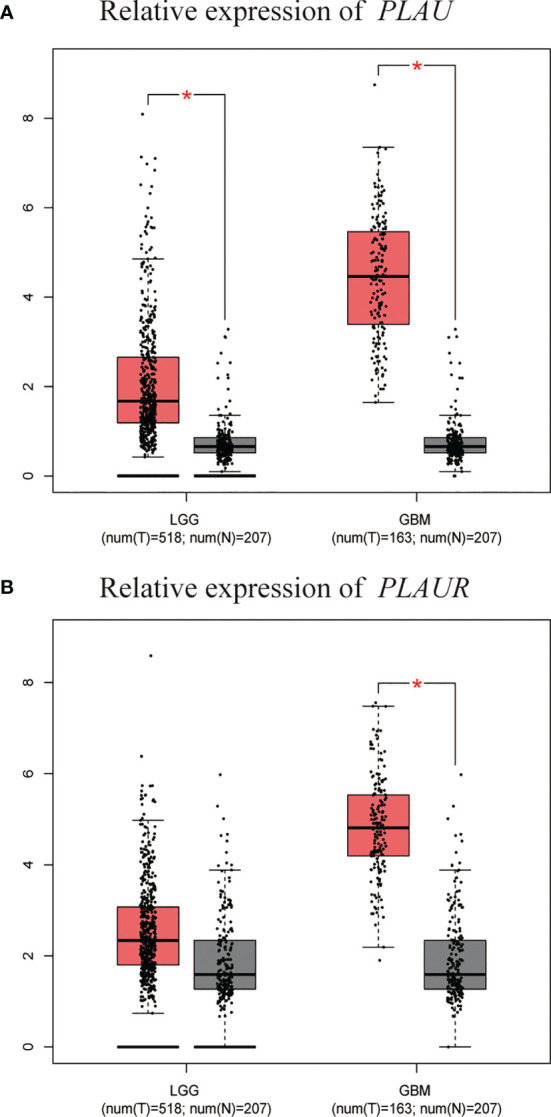
Transcriptional expression of *PLAU* **(A)** and *PLAUR* **(B)** in gliomas and normal brain tissues(GEPIA). *p < 0.01.

A similar analysis was performed in GEPIA. As shown in **Figure 1**, transcription levels of *PLAU* and *PLAUR* were evidently higher in GBM and lower-grade glioma (LGG) than in normal brain tissues. **Figure 1** also indicated that the expression levels of two genes in GBM were higher than LGG.

### Association of mRNA Expression of *PLAU* and *PLAUR* With Clinicopathological Parameters in Glioma Patients

CGGA databases were retrieved for the clinical data of glioma patients, and one of the databases was acquired for further research (**Supplementary Tables 1**, **2**). The database recorded 693 cases of glioma patients (**Table 2**), including 398 males and 295 females. Among them, 422 cases were primary gliomas while 271 cases were recurrent gliomas. Except for one case without information about the WHO grade, 249 cases were diagnosed as GBM, and 443 cases were diagnosed as LGG. There was no WHO grade I case in the dataset. IDH mutation status was found in 642 cases while 1p19q status was available in 623 cases. Results of stratification and statistic analysis were shown in **Table 2**. The mRNA expressions of *PLAU* and *PLAUR* varied remarkably among different ages, tumor histology, WHO grades and tumor types, IDH-1 mutation, and 1p19q status and other clinical characteristics. The expressions of *PLAU* and *PLAUR* were evidently higher in higher WHO grade, recurrent gliomas, wild type IDH-1 and non-codeletion of 1p19q than their counterparts (p<0.0001). With regard to age, older patients had higher *PLAU*(p=0.0043) and *PLAUR*(p<0.0001) mRNA level. There was no difference between men and women in *PLAU* and *PLAUR* expression (p=0.8609, p=0.1790, respectively).

**Table 2:** Clinic characteristics of 693 glioma patients from the CGGA database.

Clinical features		Case (N)	Relative Expression of *PLAU* (Median)	P value	Relative Expression of *PLAUR* (Median)	P value
**Age**	<45	382	3.900	***0.0043***	4.130	***<0.0001***
	≥45	310	2.885		6.840	
**Gender**	Male	398	3.050	0.8609	5.095	0.1790
	Female	295	3.450		4.990	
**Histology**	Non-GBM	443	1.830	***<0.0001***	3.420	***<0.0001***
	GBM	249	9.530		12.850	
**WHO grade**	II	188	1.545	***<0.0001***	3.090	***<0.0001***
	III	255	2.220		3.840	
	IV	249	9.530		12.850	
**Tumor Type**	Primary	422	2.540	***<0.0001***	3.995	***<0.0001***
	Recurrence	271	5.090		7.460	
**IDH-1 mutation status**	Mutant	356	2.240	***<0.0001***	3.510	***<0.0001***
	Wild-type	286	8.815		11.58	
**1p19q status**	codeletion	145	1.340	***<0.0001***	2.420	***<0.0001***
	Non-codeletion	478	5.415		7.200	

Significant findings (p＜0.05) are expressed in bold and italic.

### Prognostic Value of *PLAU/PLAUR* Transcription Expression in Gliomas

**Figure 2:**
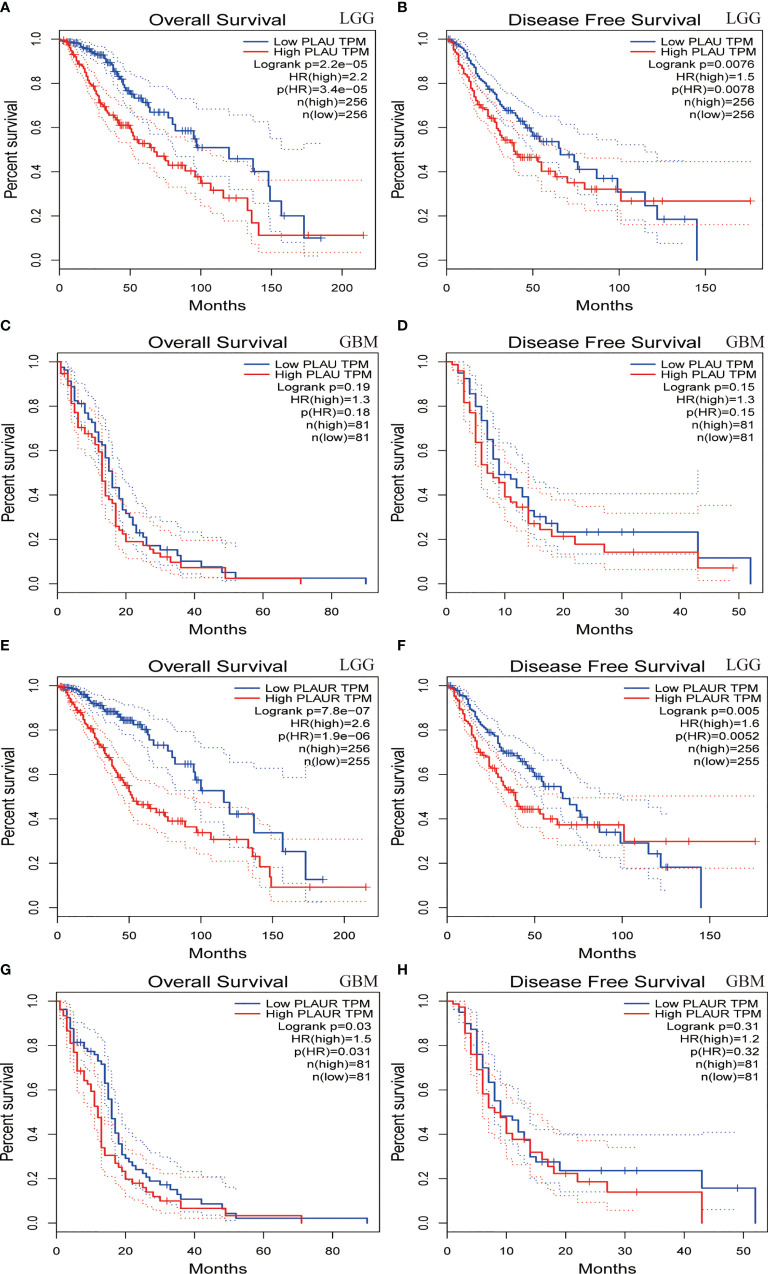
Overall survival and disease-free survival analyses based on *PLAU* and *PLAUR* expression in lower grade gliomas(LGG) **(A, B, E, F)** and GBM **(C, D, G, H)**. (GEPIA from TCGA).

**Figure 3:**
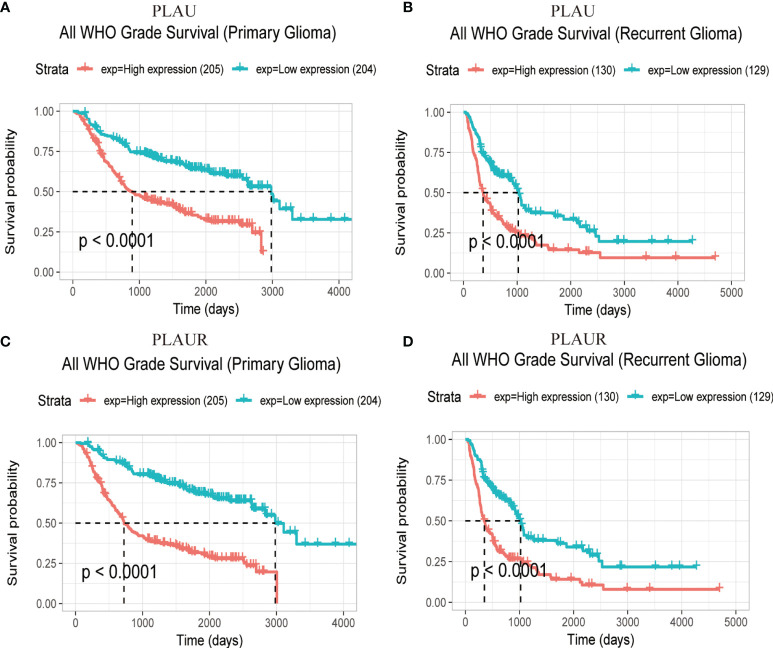
Prognostic significance of *PLAU* and *PLAUR* expression in primary gliomas **(A, C)** and recurrent gliomas **(B, D)**. (CGGA).

Prognostic statistics were retrieved from GEPIA and CGGA (**Figure 2**). In the GEPEIA database, shown in **Figures 2A, B**, high expression of *PLAU* in LGG was associated with a shorter OS(p=2.2e-5) and DFS(p=0.0076), whereas no difference was found between high and low expression of *PLAU* in OS and DFS(p=0.19, p=0.15 respectively) (**Figures 2C, D**) in GBM. In the CGGA database, high expression of *PLAU* indicated poor prognosis in WHO grade II (p=0.03) and III patients(p=0.00094) (**Figures S1A, B**). Similarly, the overall survival was unchanged with the change of *PLAU* expression level(p=0.7) (**Figure S1C**). High expression of *PLAU* predicted poor prognosis in regard of all gliomas. (p<0.0001) (**Figure 3A**).

**Figures 2E, F** demonstrated that among LGG patients, high expression of *PLAUR* resulted in shorter OS and DFS(p=7.8e-7, p=0.005 respectively), while in GBM patients, high expression of *PLAUR* predicted a shorter OS(p=0.03) (**Figure 2G**), but no significant correlation was found between high expression of *PLAUR* with shorter DFS(p=0.31) (**Figure 2H**). In the CGGA database, overall survival difference was detected between *PLAUR* high expression and *PLAUR* low expression group in WHO grade II (p=0.021) and III patients(p=0.0028) (**Figure S1G, H**), but not in GBM(p=0.2) (**Figure S1I**). Likewise, high expression of *PLAUR* indicated poor prognosis in all gliomas (p<0.0001) (**Figure 3C**).

With regard to recurrent glioma, complete data was achieved from CGGA. Among patients of WHO grade II and IV, expression of *PLAU* (p=0.84, p=0.2 respectively) (**Figures S1D, F**) and *PLAUR* (p=0.14, p=0.16 respectively) (**Figures S1J, L**) were of no significance in OS. However, high expression of *PLAU* and *PLAUR* indicated shorter OS in patients with current gliomas of WHO grade III (p=0.0041, p=0.00079 respectively) (**Figures S1E, K**). And in all recurrent patients, high expression of PLAU and PLAUR predicted poor prognosis (p<0.0001, p<0.0001 respectively) (**Figures 3B, D**).

A cox regression analysis was conducted to further confirm the independent prognostic significance of *PLAU/PLAUR* mRNA expression. As shown in **Table 3**, high mRNA expression of *PLAU* (HR=1.578, 95%CI 1.208-2.061, p=0.0008) and *PLAUR*(HR=1.38, 95%CI 1.05-1.814, p=0.0211) were independently associated with significantly shorter OS of glioma patients. At the same time, clinical variables including tumor type, WHO grade, age, IDH mutation, and 1p19q status were regarded as independent prognostic factors.

**Table 3:** Cox regression analysis of overall survival in 693 patients.

Variables		Cox Regression Analysis		
		Hazard ratio	95% CI	P value
**Tumor type**	Recurrence VS Primary	2.051	1.639-2.571	***<0.0001***
**WHO Grade**	IV VS II	4.348	2.950-6.410	***<0.0001***
	IV VS III	1.623	1.218-2.165	***0.0009***
**Gender**	Female VS Male	0.96	0.766-1.203	0.7241
**Age**	≥45 VS <45	1.381	1.093-1.748	***0.007***
**IDH mutation Status**	Wild type VS Mutation	1.567	1.185-2.070	***0.0016***
**1p19q codeletion Status**	Non-codeletion VS Codeletion	2.188	1.490-3.215	***<0.0001***
**PLAU**	High expression VS Low expression	1.578	1.208-2.061	***0.0008***
**Tumor type**	Recurrence VS Primary	2.024	1.613-2.545	***<0.0001***
**WHO Grade**	IV VS II	4.367	2.959-6.452	***<0.0001***
	IV VS III	1.631	1.215-2.188	***0.0011***
**Gender**	Female VS Male	0.959	0.765-1.202	0.7169
**Age**	≥45 VS <45	1.326	1.049-1.678	***0.018***
**IDH mutation Status**	Wild type VS Mutation	1.58	1.196-2.096	***0.0013***
**1p19q codeletion Status**	Non-codeletion VS Codeletion	2.288	1.562-3.344	***<0.0001***
**PLAUR**	High expression VS Low expression	1.38	1.05-1.814	***0.0211***

Significant findings (p＜0.05) are expressed in bold and italic.

### Genetic Alterations in *PLAU/PLAUR* and Their Association With OS of Glioma Patients

**Figure 4:**
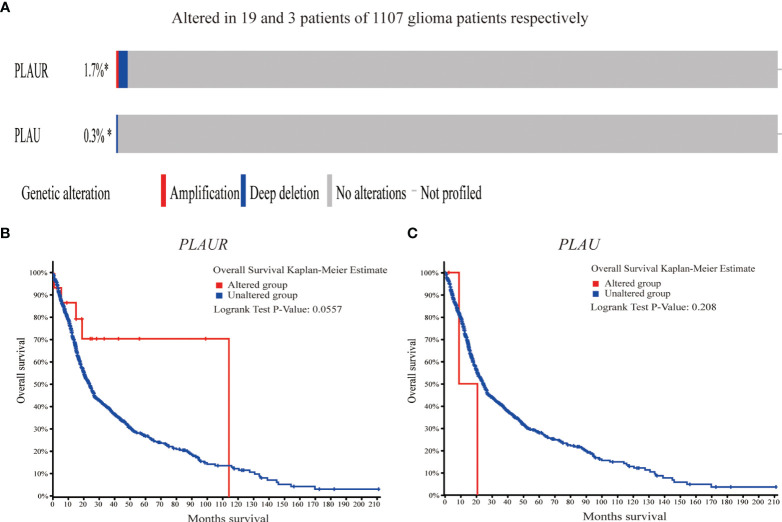
Genetic alterations of *PLAU* **(A)** and *PLAUR* and their association with OS in gliomas **(B, C)**.

As shown in **Figure 4**, genetic alterations (amplification and deep deletion) were analyzed in 1107 sequenced glioma samples, with alteration rates of 0.3% and 1.7% in *PLAU* and *PLAUR* respectively. Furthermore, survival analysis indicated that there was no significant difference between the altered group and the unaltered group in OS, considering either *PLAU* (log-rank test p=0.208) or *PLAUR*(log-rank test p=0.0557).

### Gene Set Enrichment Analysis of *PLAU/PALUR* Functional Networks in Gliomas

**Figure 5:**
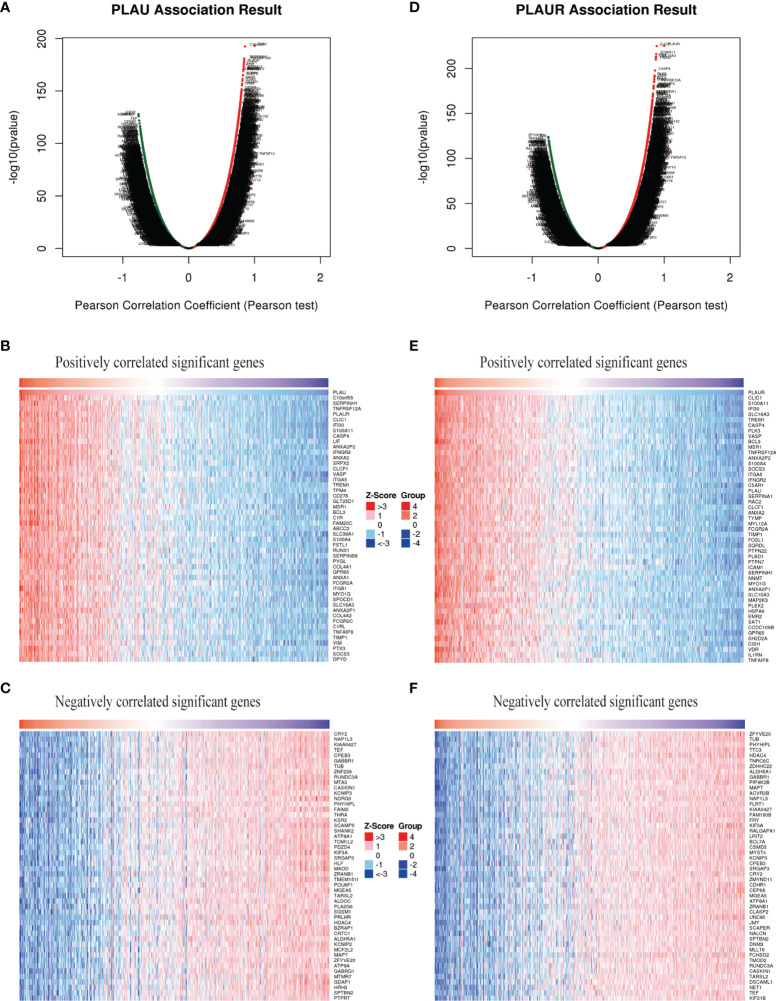
*PLAU* **(A–C)** and *PLAUR* **(D–F)** co-expression genes in gliomas (LinkedOmics).

**Figure 6:**
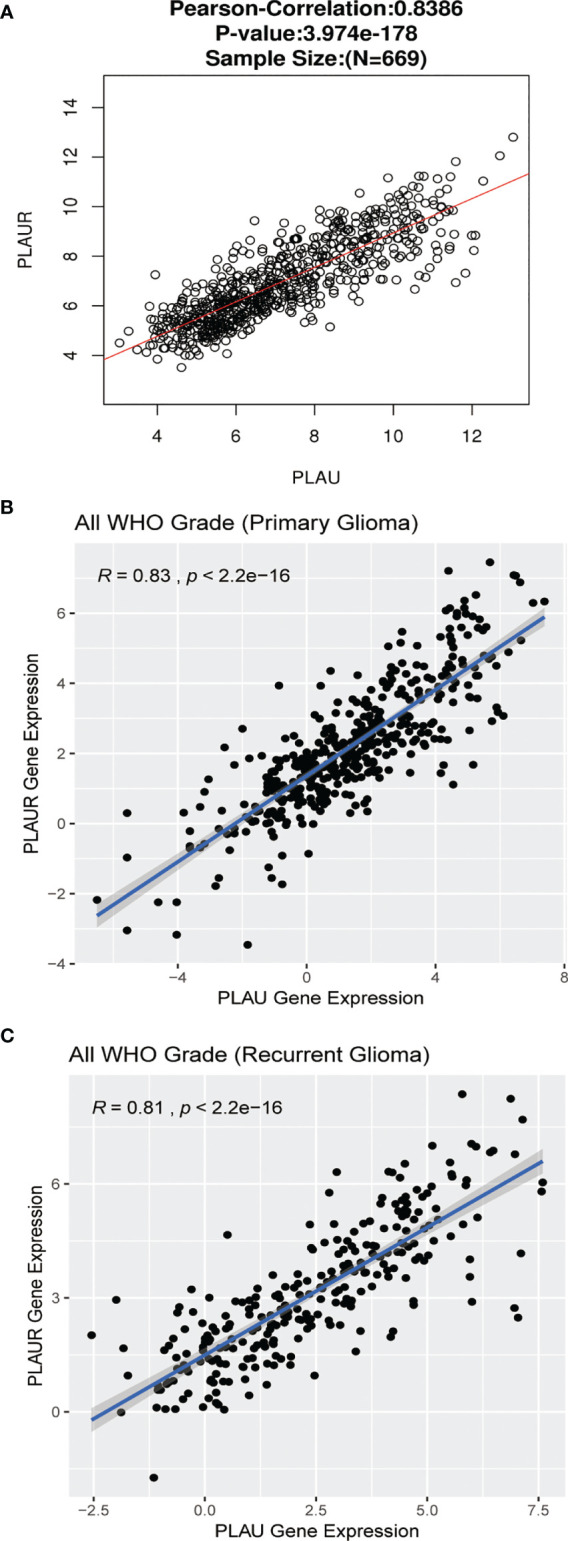
Genetic correlation of *PLAU* and *PLAUR* with Pearson correlation analysis. **(A)** Linkedomics. **(B, C)** CGGA database.

To further explore the biological meaning of *PLAU* and *PLAUR* in glioma patients, the function module of LinkedOmics was applied to analyze mRNA sequencing data from the 669 glioma patients. As shown in **Figures 5A, D**, genes that were positively or negatively correlated with *PLAU* and *PLAUR*(false discovery rate, |FDR|<0.01) were represented by dark red dots and dark green pots respectively. And the top 50 positively and negatively correlated significant genes were shown in the heat map (**Figures 5B, C, E, F**). The above-mentioned co-expressed genes were detailedly described in **Supplementary Tables 3**, **4**. A positively significant correlation of *PLAU* and *PLAUR* was found in the gene list through Pearson correlation analysis (Pearson correlation coefficient=0.8386) (**Figure 6A**). Same analysis was carried out on the previous 693 patients of the CGGA database to verify the accuracy of the result. Pearson correlation analysis indicated a high positive correlation between *PLAU* and *PLAUR* transcription expression in primary gliomas (Pearson correlation coefficient=0.83) and recurrent gliomas (Pearson correlation coefficient=0.81) (**Figures 6B, C**).

**Figure 7:**
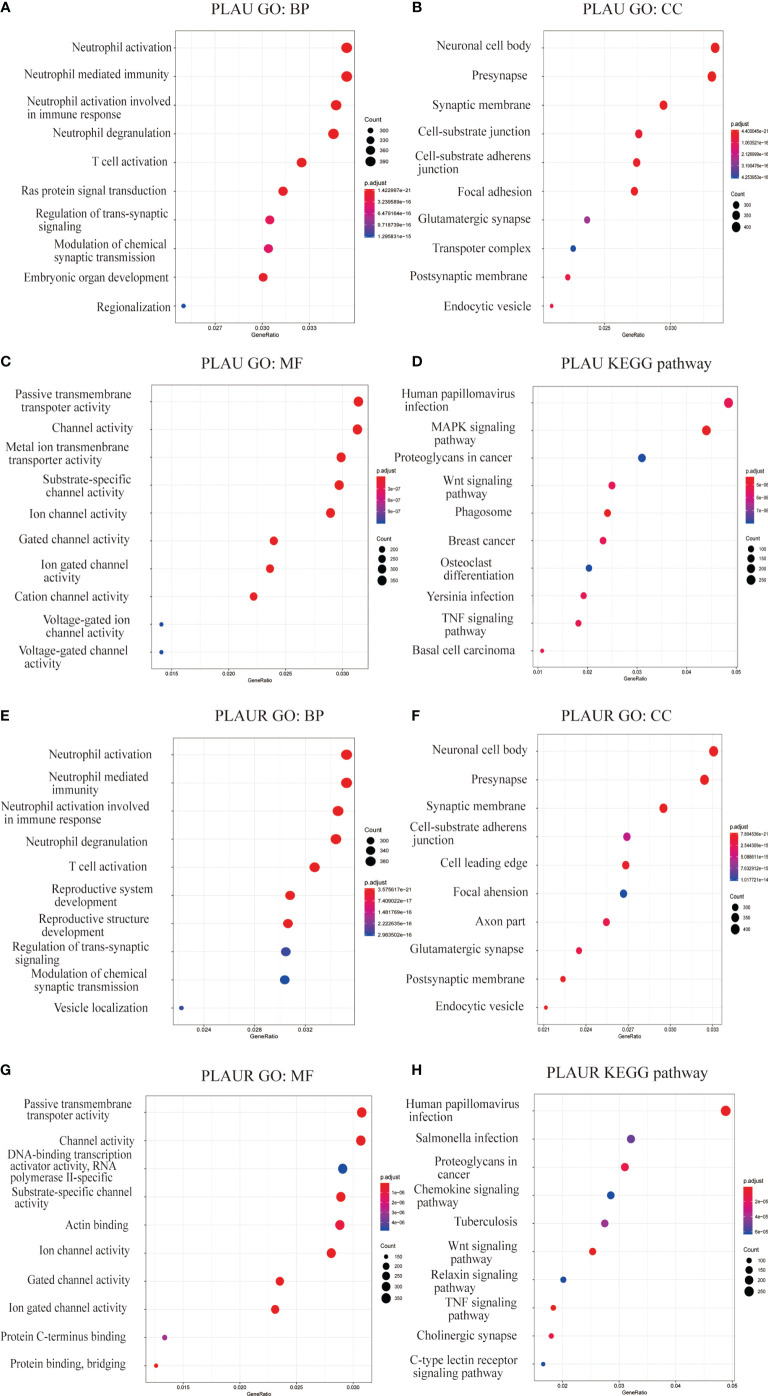
Gene set enrichment analyses of *PLAU* and *PLAUR* co-expression genes in gliomas. **(A–C, E–G)** Gene Ontology analyses including biological process (BP), cellular component (CC), molecular function (MF) of *PLAU* and *PLAUR* co-expression genes in gliomas. **(D, H)** KEGG pathway analyses of *PLAU* and *PLAUR* co-expression genes in gliomas.

Gene set enrichment analysis(GSEA) included Gene Ontology(GO) analysis and Kyoto Encyclopedia of Genes and Genomes(KEGG) pathway analysis. GO analysis indicated that *PLAU* co-expressed genes were mainly involved in the biological processing of neutrophil and T cell, and participated in the construction of the nerve-related structure and cell-substrate junction(**Figures 7A, B**). And these genes were linked to the activities of transmembrane transporter and channels (**Figure 7C**). Likewise, *PLAUR* and its co-expressed genes not only had a close relationship with immune cells but also played a crucial role in the neuronal structure, cell-substrate junction, and transmembrane activity (**Figures 7E–G**).

KEGG pathway analysis showed *PLAU* and co-expressed genes enrichment in human papillomavirus infection, MAPK signaling pathway, Wnt signaling pathway, and proteoglycans in cancer, etc.(**Figure 7D**). Whereas, PLAUR and co-expressed genes enriched in human papillomavirus infection, salmonella infection, proteoglycans in cancer, and chemokine signaling pathway, etc. (**Figure 7H**).

## Discussion

PLAU, by binding to PLAUR which is located on the extracellular surface, activates a cascade of extracellular proteases, which are involved in matrix remodeling and cell migration. As functional integrity, the PLAU-PLAUR system is supposed to not only plays a crucial role in mediating proteolysis during cancer invasion and metastasis but also participates into multiple stages of tumorigenesis (34). In recent years, high transcriptional levels of *PLAU* and *PLAUR* have been discovered in various tumors and have predicted a poor prognosis among the patients (12, 35–37). As a representative of malignancies, glioma, especially GBM, possesses common tumor characteristics including invasion, angiogenesis, epithelial-mesenchymal transition, cancer stem cell-like properties, and metastasis. Previous studies have demonstrated that these malignant features have a positive correlation with high expression of *PLAU* and *PLAUR* (16, 17, 38, 39). This study aims to evaluate the prognostic value of *PLAU/PLAUR* transcription expression in glioma and to explore how these pairs of genes affect the generation and progression of glioma.

Due to the small sample size and uncertainty of sample quality, we abandoned the traditional research methods and turned to data-rich online tools including Oncomine, GEPIA, and CGGA, etc. We can acquire a more authentic and comprehensive perspective using different online databases, which provide more information on tumors, both supplementing and verifying each other. The data of patients and gliomas are mainly from TCGA and CGGA; the former involves the Western region while CGGA only includes Chinese patients’ information. By the way, in consideration of the rarity of WHO I glioma, we usually regard LGG as WHO II and III glioma.

*PLAU* and *PLAUR* transcriptional expressions are found significantly higher in glioma compared with normal brain tissue, and are more obvious in high-grade gliomas. The result is coincident with previous studies (21, 22). The transcription expression even has a close relationship with IDH-1 mutation and 1p19q status. Higher expression occurred in wild-type IDH-1 and 1p-19q non-codeletion glioma. This demonstrates that high malignancy leads to high expression of *PLAU/PLAUR* since wild-type IDH-1 and 1p19q non-codeletion are features of malignancy (40). This can also explain why GBM has the highest expression. With regard to the survival analysis, there is high mRNA level of *PLAU/PLAUR* in primary and recurrent gliomas as a whole. After stratification of WHO grade, only LGG predicts poor prognosis. In GBM and part of recurrent gliomas, the prognostic value is questionable. There might be two possible explanations. On the one hand, high heterogeneity of malignant glioma leads to the diversity of OS; on the other hand, the sample size of GBMs and recurrent gliomas is relatively small. The results of Cox regression analysis further demonstrate the independent prognostic significance of *PLAU/PLAUR* in glioma. Interestingly, the two genes are similarly in the shape of the Kaplan-Mier plots, especially in the CGGA database. High Pearson correlation efficiency between *PLAU* and *PLAUR* indicates that the two genes share the same prognosis significance and could be integrated into one prognostic prediction model.

In view of the high incidence of genetic mutations in glioma, cBioPortal is used to evaluate the gene alteration frequency and whether the alteration affects overall survival (41). As shown in **Figure 3**, the gene alteration frequencies of 2 genes are negligible, without impact on overall survival. Another TCGA glioma cohort is retrieved in cBioPortal, and the results remain the same. It means *PLUR/PLAUR* and transcriptional expression are less affected by genetic mutations which usually cause phenotypic changes.

Extracellular matrix(ECM) breakdown is an important step for cell invasion and metastasis, and ECM proteinases such as PLAU/PLAUR system plays a key role in this process. Growing evidences indicate that down-regulation of *PLAU* and *PLAUR* attenuate the ability of tumor cells to invade and metastasize, but what triggers the regulation remains a critical question (6). DNA methylation and MicroRNAs are speculated to be the effective transcriptional regulatory ways (42).

*PLAU/PLAUR* co-expression network is constructed to further analyze their role in cell function and signaling pathway. *PLAU* and *PLAUR* share a lot of similarities in the gene set enrichment analysis. They both had a close relationship with immune cells like T cell and neutrophil during the biological process. The PLAU-deficient mice failed to develop T cells and macrophages and thus dying of bacterial infection was once reported (43). It is also indicated in the previous research that *PLAU* mediates regulatory T cells suppressor function through STAT5 and ERK signaling pathways, whereas regulatory T cells participates in complicated immunoreaction in glioma microenvironment (44, 45). That *PLAUR* plays a role in lymphocyte migration is also reported (46). At the same time, the close relationship between the PLAU/PLAUR expression and immune cells might be a key factor in the development of rheumatoid arthritis (7). Except for some inflammation-related signaling pathways, several tumor-related signaling pathways have a relationship with *PLAU* and *PLAUR* co-expression networks. KEGG pathway analysis suggests that *PLAU* and *PLAUR* are mainly involved in the proteoglycan-related cancer signaling pathway, Wnt signaling pathway, and TNF signaling pathway. Proteoglycan-related signaling pathway contributes to the biology of various types of cancer including proliferation, adhesion, angiogenesis, and metastasis, thus affecting tumor progress (47, 48). The Wnt signaling pathway and TNF signaling pathway are also involved in tumorigenesis of various tumors. Inhibition of expression of PLAU/PLAUR might blockade these crucial cancer-related pathways, and plays an anti-tumor role in gliomas. Gondi et al. revealed that bicistronic adenoviral construct targeting PLAU and PLAUR inhibited invasiveness and tumorigenicity in GBM cell lines (15). Other researches also indicated suppression of PLAU and PLAUR could attenuate the ability of glioma cells *in vivo* and *vitro* (16, 18, 20, 49).

There are some limitations in this study. First, though some recurrent gliomas are found with high expression of *PLAU* and *PLAUR* that predict poor prognosis, it is unknown whether the two genes are involved in the recurrence of glioma. Second, the data are insufficient to access the potential diagnostic and therapeutic roles of *PLAU/PLAUR* in glioma. Third, more detailed clinic data including tumor size, location, the extent of resection, and even pathological and imaging information are needed to stratify and analyze in a more accurate way. Fourth, these analyses are almost limited to the mRNA level, lacking evidence from protein level and functional studies make it less persuasive. Fifth, it is hard to know how PLAU and PLAUR expression can be involved in the glioma formation, or their expression is a consequence of the glioma development. These limitations should be addressed in follow-up studies.

In conclusion, our current study indicated that overexpression of *PLAU* and *PLAUR* is associated with poor prognosis in primary and recurrent glioma patients, especially in LGG. Overexpression of *PLAU* and *PLAUR* is regarded as independent prognostic factors for shorter OS of glioma patients through Cox regression analysis. Moreover, Gene co-expression network analysis enlightens us that immune therapy and specific cancer-related signaling pathway blocking by targeting *PLAU/PLAUR* might be a new idea for treating glioma.

## Data Availability Statement

The datasets generated and analyzed during the current study are available in the Oncomine, GEPIA, LinkedOmics, cBioPortal, and CGGA databases.

## Author Contributions

Study design: YL and JL. Database retrieve: JL and HF. Statistical Analysis: JL, YX, and HF. Result interpretation: YL, JL, and XZ. Manuscript preparation: JL and XZ. Text correction: YL and YX. All authors contributed to the article and approved the submitted version.

## Funding

This study was supported by the Key Research and Development Item from the Department of Science and Technology of Sichuan Province, China (No.2017SZ0006).

## Conflict of Interest

The authors declare that the research was conducted in the absence of any commercial or financial relationships that could be construed as a potential conflict of interest.

## Publisher’s Note

All claims expressed in this article are solely those of the authors and do not necessarily represent those of their affiliated organizations, or those of the publisher, the editors and the reviewers. Any product that may be evaluated in this article, or claim that may be made by its manufacturer, is not guaranteed or endorsed by the publisher.

## Supplementary Material

Supplementary Figure 1 Prognostic significance of *PLAU* and *PLAUR* expression in gliomas with stratification of WHO grade(CGGA).

Supplementary Table 1 Clinical characteristics with PLAU(CGGA).

Supplementary Table 2 Clinical characteristics with PLAUR(CGGA).

Supplementary Table 3 Co-expression genes of PLAU(LinkedOmics).

Supplementary Table 4 Co-expression genes of PLAUR(LinkedOmics).
