# Supplementary figures and images for: Prognostic Significance and Gene Co-Expression Network of PLAU and PLAUR in Gliomas
Source: Front Oncol. 2022 Jan 11;11:602321. doi: 10.3389/fonc.2021.602321 (PMC8787124; doi:10.3389/fonc.2021.602321)

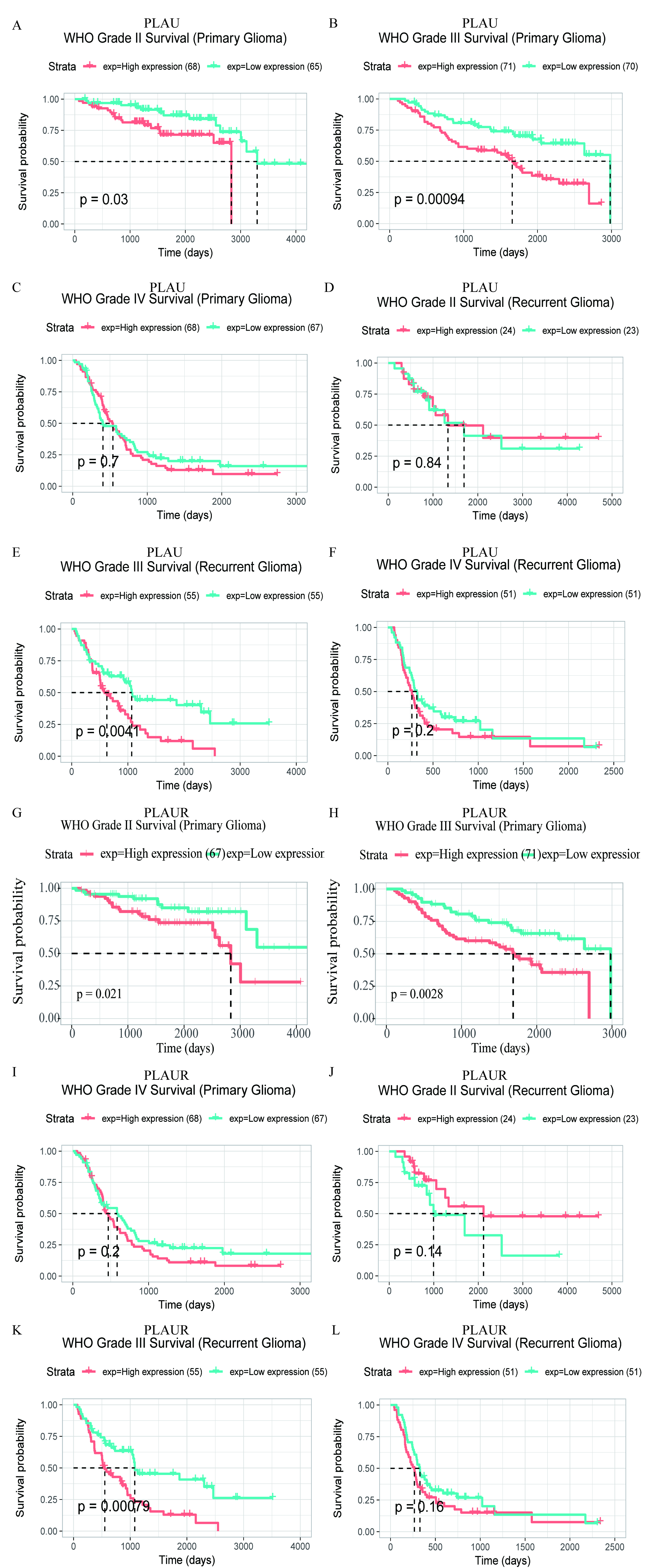

Supplement: Supplementary Figure 1 — Prognostic significance of PLAU and PLAUR expression in gliomas with stratification of WHO grade(CGGA). [file Image_1.tif]
